# Molecular Characterization and Functional Analysis of a Ferritin Heavy Chain Subunit from the Eri-Silkworm, *Samia cynthia ricini*

**DOI:** 10.3390/ijms18102126

**Published:** 2017-10-14

**Authors:** Hai-Zhong Yu, Shang-Zhi Zhang, Yan Ma, Dong-Qiong Fei, Bing Li, Li-Ang Yang, Jie Wang, Zhen Li, Azharuddin Muhammad, Jia-Ping Xu

**Affiliations:** School of Life Sciences, Anhui Agricultural University, Hefei 230036, China; yuhaizhong1988@163.com (H.-Z.Y.); 18755148780@163.com (S.-Z.Z.); matafeiyan2016@163.com (Y.M.); FDq1016@163.com (D.-Q.F.); libing2504@sina.com (B.L.); YLAPYF@163.com (L.-A.Y.); wangjie_3001@163.com (J.W.); Alexli6052@163.com (Z.L.); azhar.pharmacist1000@gmail.com (A.M.)

**Keywords:** ferritin, *Samia cynthia ricini*, immune response, iron binding capacity, anti-oxidation activity

## Abstract

Ferritins are conserved iron-binding proteins that are primarily involved in iron storage, detoxification and the immune response. Despite the importance of ferritin in organisms, little is known about their roles in the eri-silkworm (*Samia cynthia ricini*). We previously identified a ferritin heavy chain subunit named ScFerHCH in the *S. c. ricini* transcriptome database. The full-length *S. c. ricini* ferritin heavy chain subunit (*ScFerHCH*) was 1863 bp and encoded a protein of 231 amino acids with a deduced molecular weight of 25.89 kDa. Phylogenetic analysis revealed that ScFerHCH shared a high amino acid identity with the *Bombyx mori* and *Danaus plexippus* heavy chain subunits. Higher *ScFerHCH* expression levels were found in the silk gland, fat body and midgut of *S. c. ricini* by reverse transcription quantitative polymerase chain reaction (RT-qPCR) and Western blotting. Injection of *Staphylococcus aureus* and *Pseudomonas aeruginosa* was associated with an upregulation of *ScFerHCH* in the midgut, fat body and hemolymph, indicating that *ScFerHCH* may contribute to the host’s defense against invading pathogens. In addition, the anti-oxidation activity and iron-binding capacity of recombinant ScFerHCH protein were examined. Taken together, our results suggest that the ferritin heavy chain subunit from eri-silkworm may play critical roles not only in innate immune defense, but also in organismic iron homeostasis.

## 1. Introduction

Iron is an essential nutrient element for almost all living organisms, including hosts and invaders [1], because of its important role in various biological processes, including growth and differentiation, oxygen transport and storage, DNA synthesis and the cell cycle. From *Archaea* to man, these organisms are dependent on iron for survival. Mammalians cells require sufficient amounts of iron to satisfy metabolic needs [2,3,4,5]. In addition, iron is also involved in cuticle formation, tanning, melanization and wound healing [6]. In spite of its requirement and these features, it is also a potent toxin because high levels of free iron in the body lead to the oxidative damage of proteins, lipids, DNA, as well as potentiating serious effects on the integrity of cell membranes and increasing the risks of cell damage [7,8]. The tight regulation of iron metabolism maintains a balance between its benefits and toxic effects, and it is accomplished by the iron-binding proteins (IBPs) such as transferrin and ferritin.

Ferritin is a 450-kDa storage protein that was first identified in the midgut epithelial cells of *Philaenus spumarius* [9]. This protein is ubiquitous in a wide variety of organisms, including bacteria, fungi, invertebrates and vertebrates, showing several conserved features [10,11,12,13]. Ferritin is involved in multiple functions, such as iron storage and release [14], inflammation [15], and developmental regulation [16]. The typical ferritins are composed of 24 subunits, which fold in a four-helical bundle that assembles into a spherical protein shell with four-, three-, two-point symmetry [17]. The shell can sequester up to 4500 Fe (III) ions, which communicate with the solvent via six hydrophilic channels on the threefold symmetry. Of course, iron uptake requires a specific catalytic site known as the ferroxidation center, which is a carboxylate-bridged di-iron center situated in the subunit four-helix bundle. It can be oxidized to Fe (III) through multiple reactions involving dioxygen or peroxides [18]. Ebrahimi et al. observed that a site in the di-iron catalytic center can control the distribution of Fe (II) among subunits of human H-type ferritin (HuHF) and *Pyrococcus furiosus* ferritin (PfFtn) differently. The third transient site, known as site C, has been identified and played an important role as a gateway to the ferroxidase center in eukaryotes, bacteria and archaeal ferritins. Furthermore, a conserved tyrosine can act as a single-electron molecular capacitor to facilitate the oxidation of Fe(II) for eukaryotic and bacterial ferritin. In addition, using a combination of binding experiments and isotopically labeled ^57^Fe(II), the results suggested a unifying mechanism in which the Fe(III)-O-Fe(III) unit residues in the ferroidase center until it is sequentially displaced by Fe(II) between PfFtn and HuHF [19,20,21].

Ferritin has been identified in various insect species. The insect ferritins are composed of two types of subunits: heavy chain homologs (HCHs) and light chain homologs (LCHs) [22]. In contrast to mammals, most insect ferritins are secreted proteins that are found exclusively in the secretory pathways of cells and in the hemolymph [23]. Otho et al. identified a silkworm ferritin one heavy chain that played a crucial role in regulating the haemolymph iron homeostasis in defense against bacterial infection [24]. Georgeva et al. reported the heavy chain homologue (HCH) of the *Drosophila melanogaster* ferritin subunit using Northern blot analysis. The results revealed that synthesis of ferritin HCH subunit mRNAs may be important under conditions of iron overload due to the absence of an iron responsive element (IRE) [25]. In *Aedes aegypti*, the ferritin heavy-chain gene was induced by blood feed, suggesting that ferritin might serve as a cytotoxic protector against the oxidative challenge of the blood meal [26]. Furthermore, members of the ferritin family have been identified in a variety of fishes and shellfishes, including *Haliotis diversicolor* [27], *Stichopus monotuberculatus* [28] and *Chlamys farreri* [10]. However, the structure and functions of the ferritin heavy chain subunit in *S. c. ricini* have not been characterized.

*Samia cynthia ricini* (Lepidoptera: Saturniidae) is a commercial silk-producing insect, originating from India, China and Japan [29]. It is a holometabolous insect, with four stages in its life history, including egg, larva, pupa and adult. Hence, the eri-silkworm can provide a good model for molecular biology research [30]. In addition, individuals of *S. c. ricini* are confronted with many potential pathogenic microorganisms and heavy metals in their natural habitat. Therefore, we focused on the role of ferritin in the immune response, antioxidation and the regulation of iron homeostasis [18,28].

In this study, we identified the full-length cDNA of the ferritin heavy chain subunit from the database of the eri-silkworm transcriptome and investigated its expression pattern in different tissues. In addition, the expression levels following challenges with *Staphylococcus aureus* and *Pseudomonas aeruginosa*, as well as the biological function of recombinant ferritin protein were also analyzed. Our results suggest that ScFerHCH is likely to play an important role in iron storage, antioxidation, and innate immune defense against pathogens in *S. c. ricini*. These results will lay a foundation for further characterization of ScFerHCH functions.

## 2. Results

### 2.1. Identification of the ScFerHCH Gene and Sequence Analysis

**Figure 1:**
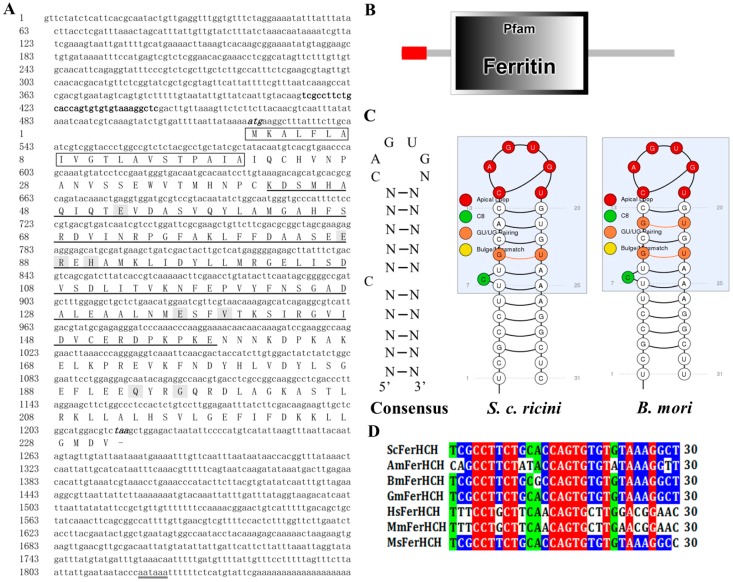
(**A**) Complete nucleotide and deduced amino acid sequence of the *S. c. ricini* ferritin heavy chain subunit (*ScFerHCH*) gene. Numbers on the left side represent nucleotide and amino acid positions. The putative IRE in the 5′-UTR is shown in black bold, and the polyadenylation signal in the 3′-UTR is double underlined. The initiation codon (ATG) and termination codon (TAA) are indicated in black italics. The signal peptide is represented in the black box. The ferritin domain is highlighted in a single line, and the eight metal ligands are shaded; (**B**) Structural domain of ScFerHCH predicted using the SMART program. The red box indicates the signal peptide and the black box represents the ferritin domain; (**C**) Predicted IRE stem-loop structures of the ferritin heavy chain subunit from *S. c. ricini* (Accession no. 2045712 ) and *B. mori* (Accession no. AK386476); (**D**) Alignment of IREs of ScFerHCH with other insect ferritin. The conserved nucleotides for IREs were marked in red. Identical amino acids are highlighted in green, similar amino acids are highlighted in blue.

The *ScFerHCH* gene (GenBank Accession No: 2045712) was identified from the transcriptome database of *S. c. ricini*. The cDNA sequence has a predicted start codon at nucleotide 522 and a stop codon at nucleotide 1215. The deduced open reading frame of 696 bp encodes a putative protein of 231 amino acid residues with an estimated molecular mass of 25.89 kDa and a predicted isoelectric point of 5.67. A polyadenylation signal (AATAAA) is located 18 bp upstream of the poly-A tail. A putative IRE, with the conserved 5′-CAGUGU-3′ loop at the top is present in the 5′-UTR of *ferritin* cDNA (Figure 1A,C). Eight amino acid residues (Glu52, Glu87, Arg88, His90, Glu136, Val139, Gln193 and Gly196) were identified that are known as metal ligands associated with the metal-binding site in the ferroxidase center [31]. In addition, the first 20 residues at the N-terminus of ScFerHCH contain a sequence (MKALFLAIVGTLAVSTPAIA) that may act as a signal peptide for secretion (Figure 1A). Conserved domain prediction using SMART software indicated that the ScFerHCH protein contains the ferritin domain (42–158) (Figure 1B), which could bind a mineral core of hydrated ferric oxide, and form a multi-subunit protein shell that encloses the former and assures its solubility in an aqueous environment [32].

### 2.2. Homology Analysis and Phylogentic Analysis

**Figure 2:**
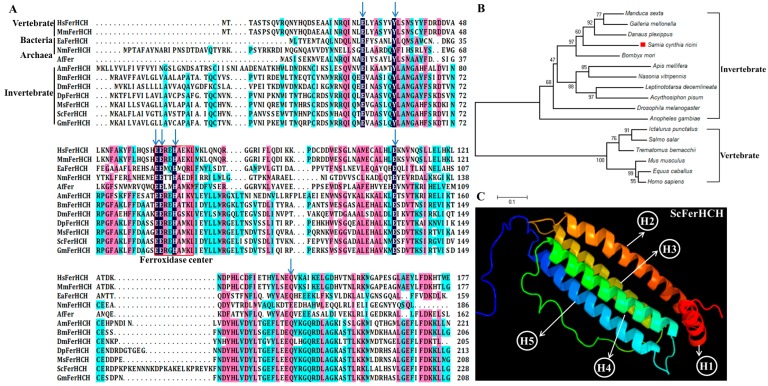
(**A**) Sequence alignment of the ScFerHCH protein with its homologs from different species. The conserved amino acid residues are highlighted in black and similar amino acid residues are labelled in pink. The conserved amino acid residues of the ferroxidase center are marked by the red box. The residues required for ferroxidase activity are indicated with blue arrows above the sequences; (**B**) Phylogenetic relationships of ferritin heavy chain subunit in different species using the neighbor-joining method with a bootstrap value of 1000; (**C**) Predicted tertiary structure of the ScFerHCH protein using the phyre2 online software. Red is the N-terminus, and blue is the C-terminus. The ScFerHCH protein contains four long α-helices (H2–H5) and one short α-helix (H1).

Multiple alignment of the Ferritin amino acid sequences of *S. c. ricini* with other known insects was performed using DNAMAN software. The ScFerHCH protein sequence has 65%, 64%, 59%, 58%, 45% and 43% identify with those from *Manduca sexta*, *Galleria mellonella*, *Danaus plexippus*, *Bombyx mori*, *Drosophila melanogaster*, and *Apis mellifera*, respectively (Figure 2A). Sequence alignment and prediction of functional domains by BLASTP revealed that it has a conserved ferrihydrite nucleation center.

In order to investigate the evolutionary relationships between ScFerHCH and those of other insects, a phylogenetic tree was constructed by the neighbor-joining method (Figure 2B). The sequences could be classified into two main clades with high bootstrap support, corresponding to invertebrates and vertebrates. The results showed that ScFerHCH had a close genetic distance with *D. plexippus* and *B. mori*.

By using the online phyre2 software, the tertiary structure of ScFerHCH was predicted. ScFerHCH contains one short α-helix (H1) and four long α-helices (H2-H5) (Figure 2C).

### 2.3. Recombinant Protein Expression, Purification and Antibody Prepartion

**Figure 3:**
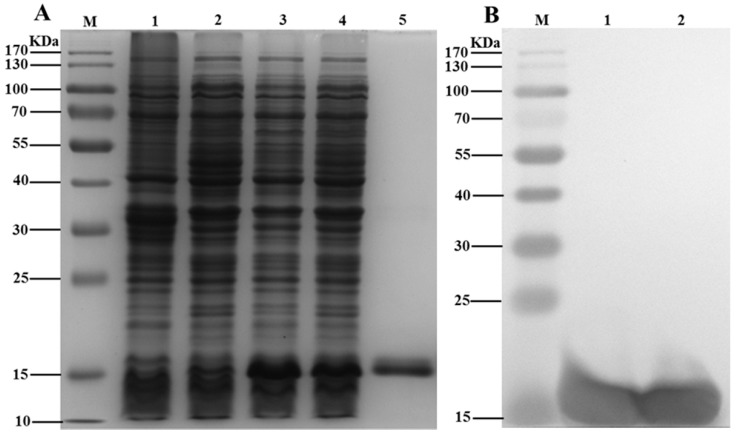
(**A**) Analysis of recombinant ScFerHCH protein using SDS-PAGE. M: molecular weight markers. Lane 1: blank control without insert. Lane 2: negative control without induction. Lanes 3–4: induced expression under IPTG concentrations of 0.6 mM and 0.8 mM. Lane 5: purified recombinant ScFerHCH protein; (**B**) Western blot analysis of recombinant His-tagged ScFerHCH protein identified by anti-His antibodies. M: molecular weight markers. Lanes 1–2: recombinant ScFerHCH protein.

In order to analyze the function of ScFerHCH protein, recombinant His-tagged ScFerHCH (ferritin domain) was expressed using a prokaryotic expression system. The recombinant protein (ScFerHCH) with a molecular mass of approximately 13.1 kDa was detected by SDS-PAGE (Figure 3). The target protein band was confirmed by Western blotting using anti-His antibodies (Figure 3), indicating that the recombinant ScFerHCH protein was successfully expressed in *E. coli* cells. The recombinant proteins were purified under denaturing conditions and used for antibody preparation. The titer of antibody was approximately 1:60,000 as determined by ELISA.

### 2.4. Tissue Distribution of the ScFerHCH Gene

**Figure 4:**
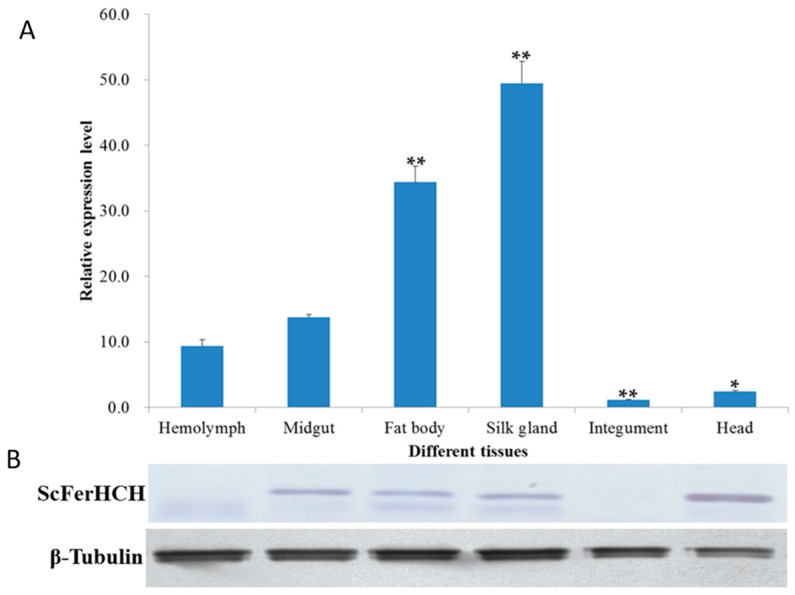
Expression profiles of *ScFerHCH* in different tissues of the fifth instar larvae of *S. c. ricini*. (**A**) The mRNA levels of *ScFerHCH* as measured by RT-qPCR. Data were normalized using *S. c. ricini β-actin* and are represented as the means ± standard errors of the means from three independent experiments. Relative expression levels were calculated using the 2^−∆∆*C*t^ method. Statistical analysis was performed using SPSS software. The significant differences are indicated by * (*p* < 0.05) or ** (*p* < 0.01); (**B**) Western blot analysis of ScFerHCH proteins in different tissues of *S. c. ricini* using β-tubulin used as an internal reference.

The tissue distribution of *ScFerHCH* was determined in the hemolymph, midgut, fat body, silk gland, integument and head of *S. c. ricini* by characterizing the expression profiles of *ScFerHCH* by RT-qPCR and Western blot analysis. The *ScFerHCH* gene was constitutively expressed in all of the examined tissues, with higher expression levels in the silk gland, followed by the fat body and midgut (Figure 4). The expression level of *ScFerHCH* in the silk gland was 5.3 times that of the hemolymph; its expression in the fat body was 3.7 times that of the hemolymph. The lowest expression levels were observed in the integument and head. Western blotting analysis showed that ScFerHCH had relatively higher expression levels in the head, followed by relatively low expression levels in the hemolymph and integument (Figure 4).

### 2.5. Expression Profiles of ScFerHCH after Challenge with Pathogens

**Figure 5:**
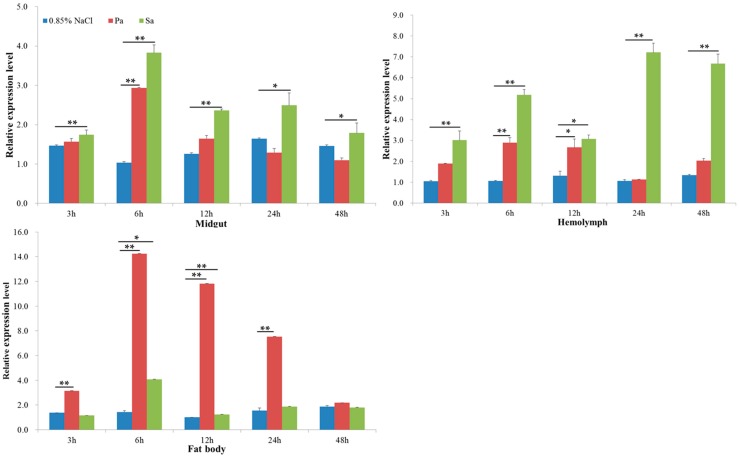
The expression levels of ScFerHCH in the midgut, fat body and hemolymph after injection with *P. aeruginosa* (Pa) and *S. aureus* (Sa) at 3, 6, 12, 24 and 48 h. Sterile 0.85% NaCl was used as a control. Relative expression levels were calculated using the 2^−^^∆∆*C*t^ method. Statistical analysis was performed using the SPSS software. The significant differences are indicated by * (*p* < 0.05) or ** (*p* < 0.01).

**Figure 6:**
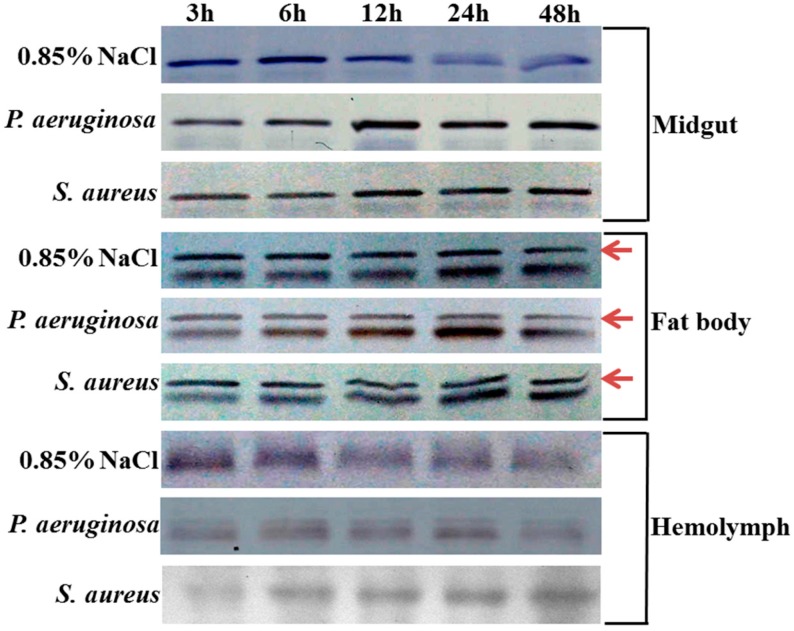
Protein expression profiles of ScFerHCH in the midgut, fat body and hemolymph post challenging by *P. aeruginosa* (Pa) and *S. aureus* (Sa) at 3, 6, 12, 24 and 48 h. Sterile 0.85% NaCl was used as a control. The red arrows indicated target band.

The expression profiles of *ScFerHCH* were examined in the midgut, fat body and hemolymph in response to pathogens using RT-qPCR and Western blotting analysis at different times post injection. A clear time-dependent expression pattern of *ScFerHCH* was observed (Figure 5). At 0–3 h after *P. aeruginosa* challenge, the expression of *ScFerHCH* was obviously up-regulated and reached a maximum level at 6 h post-challenge in the fat body. However, the expression levels of *ScFerHCH* showed no significant change in the midgut and hemolymph at 3 h after *P. aeruginosa* challenge. As time progressed, *ScFerHCH* expression levels decreased gradually. At 0–3 h after *S. aureus* challenge, *ScFerHCH* expression levels were significantly higher than the control levels in the midgut and hemolymph. At 6 h after *S. aureus* challenge, the *ScFerHCH* expression levels reached a maximum in the midgut and fat body and then dropped gradually. However, the *ScFerHCH* expression levels increased sharply at 24–48 h in the hemolymph after *S. aureus* challenge. The ScFerHCH transcription profiles correlated with ScFerHCH translational levels as detected by Western blotting of the midgut, fat body and hemolymph samples after pathogen challenge (Figure 6).

### 2.6. Iron Chelating Assay

**Figure 7:**
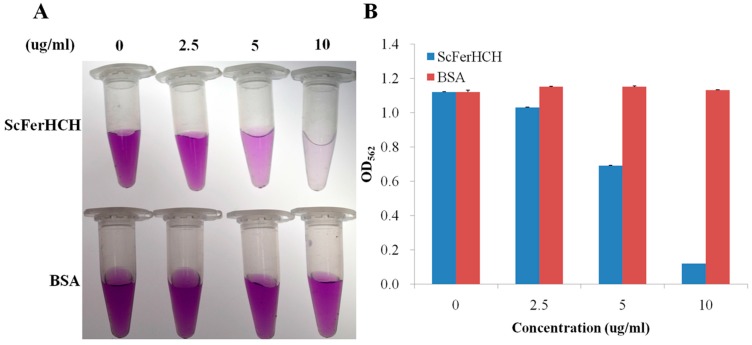
Iron-chelating activity of purified ScFerHCH. (**A**) The color reaction in different concentration of purified ScFerHCH. (**B**) Detection of iron-chelating activity in different concentration of purified ScFerHCH. The X-axis represents the concentration of recombinant ScFerHCH and bovine serum albumin (BSA). The Y-axis indicates the absorbance at 562 nm after the iron-chelating reactions.

Iron chelating assays were performed to examine whether the recombinant ScFerHCH protein could bind iron. As shown in Figure 7, with an increasing concentration of the purified ScFerHCH, the purple color became gradually thinner and the OD_562_ value also showed a decreasing trend. However, there was no significant change in the presence of bull serum albumin (BSA). The iron chelating assay indicated that the purified recombinant ScFerHCH had an iron-binding capacity in a concentration-dependent manner.

### 2.7. H_2_O_2_ Tolerance Bioassay

**Figure 8:**
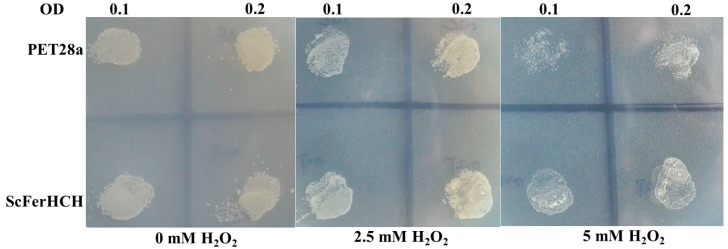
H_2_O_2_ tolerance of BL21 (DE3) *E. coli* containing the pET28a vector expressing recombinant ScFerHCH protein. Serial dilutions of cells were plated on LB agar containing different concentrations of H_2_O_2_ and incubated for 12 h at 37 °C.

As shown in Figure 8, cells expressing either control or recombinant ScFerHCH grew equally well in the LB medium without H_2_O_2_. The number of bacteria had no obvious change between the OD value equal to 0.1 and 0.2. With increasing concentrations of H_2_O_2_, the growth of *E. coli* cells was inhibited. However, when the concentration of H_2_O_2_ in the medium reached to 5 mM, the growth of *E. coli* cells with empty PET-28a plasmid, but not those with recombinant ScFerHCH plasmid, was strongly inhibited. The results suggested that the recombinant ScFerHCH protein possessed tolerance to H_2_O_2_.

## 3. Discussion

Ferritin is an iron-binding protein that is made up of 24 subunits forming a hollow spherical shell that is involved in the transport and storage of iron during iron metabolism for most species [22]. It has been widely reported in vertebrates and invertebrates in recent years. The molecular weight of ferritin for most species cannot be more than 480 kDa. However, there are some exceptions in insects or archaea. Dunkov et al. reported that the molecular weight of *M. sexta* ferritin was more than 669 kDa [33]. In addition, Ebrahimi et al. also observed that *P. furiosus* archaeoferritin can form oligomeric forms of more than 480 kDa [34]. The phylogenetic tree analysis revealed that *S. c. ricini* ferritin had a close genetic distance to *M. sexta* (Figure 2B). Therefore, we speculated that the molecular weight of *S. c. ricini* ferritin might be more than 669 kDa. The structure of *S. c. ricini* ferritin needs to be further studied. In mammals, extracellular iron is transported by transferrin, while cellular iron is stored in ferritin [35]. Furthermore, mammalian ferritin can be divided into three subgroups: mitochondrial, cytoplasmic, and serum ferritins [17]. Unlike in mammals, insect ferritins are mostly secreted proteins. In this study, a ferritin heavy chain subunit (ScFerHCH) was identified from the eri-silkworm. It possessed a signal peptide composed of 20 amino acid residues, suggesting that it was a secreted protein (Figure 1A), similar to those of other insect ferritins. In *Glossina morsitans*, both putative GmmFer1HCH and GmmFer2HCH proteins have regions of signal peptides [36]. In *Bombus ignitus*, BiFerHCH also contains a putative signal peptide [37]. The signal peptide sequences identified in ferritins indicates that ferritin serves to not only store, but also to transport iron as a secreted protein. Ferritin has ferroxidase activity due to the presence of the metal-binding site in the ferroxidase center. For ScFerHCH, eight amino acid residues were identified in the metal-binding site in the ferroxidase center. In *Stichopus monotuberculatus*, seven residues (Glu25, Tyr32, Glu59, Glu60, His63, Glu105 and Gln139) in the ferroxidase center indicated iron-binding capability. On the other hand, a putative IRE with the conserved CAGUGU sequence was identified in the *S. c. ricini* heavy chain subunit, which was conserved between *S. c. ricini* and *B. mori* (Figure 1C). In previous research, the interaction of the IRE with iron-regulatory proteins (IRPs) has revealed a fascinating and exquisite system for controlling iron homeostasis in mammalian cells [38]. The position of the IRE relative to the 5′-UTR mRNA cap structure has been shown to be a crucial factor for regulating translation [39]. Together, these results suggested that ScFerHCH plays an important role in regulating iron homeostasis and translation.

The ubiquitous expression of *ScFerHCH* in all tissues of *S. c. ricini* (Figure 4) suggested that ScFerHCH might have multiple functions associated with the presence of iron in different tissues. It was reported that the mRNA expression of *M. sexta* ferritin occurred in the midgut, fat body, and hemocytes, and the midgut was the major expression site [40]. In the fat body of *A. mellifera*, the iron-rich granules were formed by the accumulation of ferritin and its degraded forms together with elements present inside the rough endoplasmic reticulum, such as phosphorus, calcium and magnesium [41]. In this study, *ScFerHCH* was highly expressed in the silk gland, fat body and midgut. The insect fat body is a dynamic tissue that is involved in various physiological and biological functions, including detoxification, developmental regulation and immunity [42]. The insect midgut is a major tissue for the metabolism of various chemicals from food. The midgut epithelium is the first physical barrier after oral intake, and contains abundant digestive enzymes needed to obtain nutrients from food, and it also contributes to the detoxification of insecticides [43]. Therefore, we speculated that ScFerHCH was primarily involved in detoxification in the eri-silkworm. Li et al. identified a ferritin from *Dendrorhynchus zhejianggensis*, indicating that worm ferritin was a promising candidate for heavy metal detoxification [44]. Liu et al. also found that the expression of a ferritin gene (*PcFer*) from *Procambarus clarkia* may be involved in immune defense and protection of *P. clarkii* against heavy metal stress [45]. To our surprise, ScFerHCH showed the highest expression level in the silk gland (Figure 4). You et al. also observed the high expression of *B. mori* ferritin one and two (*BmFer1* and *BmFer2*) in the silk gland [46]. We considered that ScFerHCH was also likely to be involved in amino acid synthesis in eri-silkworm. Additionally, overexpression of ScFerHCH in the silk gland might protect the organism from oxidative stress. Hence, high mRNA expression levels in the silk gland, fat body and midgut implied that *ScFerHCH* may be related to immune function and detoxification and may be associated with amino acid synthesis.

Iron is an essential trace element for all living organisms. However, iron is potentially toxic due to the low solubility of the stable oxidation state, Fe(III), and to the tendency to potentiate the production of reactive oxygen species (ROS) [2,32]. In this study, the purified recombinant ScFerHCH protein showed an iron-binding capacity, suggesting that eri-silkworm ferritin could sequester the excess iron to maintain the free iron at a safe concentration. The growth of many pathogenic microorganisms, including bacteria and virus, requires iron [47,48]. Therefore, eri-silkworm growth may be inhibited after pathogen infection by a competition between ferritin and the pathogens for iron. In previous research, the concentration-dependent iron-binding capacity of the silkworm ferritin one heavy chain homolog was demonstrated [24]. Ebrahimi et al. also revealed that the hyperthermophilic archaeal anaerobe PfFtn and HuHF had a unifying mechanism in which the Fe(III)-O-Fe(III) unit residues in the ferroxidase center [21]. We speculated that the ferroxidase center of ScFerHCH might play an important role in binding Fe (II). However, whether the ScFerHCH can catalyse oxidation of Fe(II) to Fe(III) requires further research. In living organisms, the oxidation of organic substrates by ferrous iron and H_2_O_2_ is called Fenton’s reaction [49]. The ROS produced by Fenton’s reaction are responsible for the oxidative damage of DNA, lipids and proteins in cellular environments [50]. Treatment of HeLa cells with H_2_O_2_ could produce ROS, whereas overexpression of ferritin could reduce the cellular levels of ROS and oxidative stress [51]. In this study, overexpressed recombinant ScFerHCH had a higher tolerance to H_2_O_2_ than the control, suggesting that the ferritin in eri-silkworms has an anti-oxidation activity and could relieve the oxidative damage.

Ferritin has a significant immune role in invertebrates. It was involved in the immune response as an acute phase reaction protein when pathogens invaded organisms [52]. Furthermore, ferritin possessed the ability to be resistant to bacteria and bind to lipopolysaccharides. In this study, ferritin expression levels were significantly in the midgut and fat body by *P. aeruginosa* and *S. aureus*, and the expression peak was reached at 6 h after bacteria injection (Figure 5). Especially in the fat body, ScFerHCH was highly expressed at 6 h after *P. aeruginosa* infection. However, the fat body expression of *BmFer1* in *B. mori* showed no obvious change after *P. aeruginosa* or *S. aureus* infection [24]. It possible that immune process functions in the fat body may differ between *S. c. ricini* and *B. mori*. At the translational level, Western blotting analysis showed that high ScFerHCH levels after *P. aeruginosa* or *S. aureus* infection at 12 h (Figure 6). ScFerHCH exhibited a delayed phenomenon at the transcriptional and translational levels. We considered that ScFerHCH may be regulated by post-translational modification, including ubiquitination and phosphorylation. Ferritin ubiquitination could be responsible for oxidative stress in the muscles of rats bearing the G93A hmSOD1 [53]. Beazley et al. found that ferritin in avian corneal epithelial (CE) cells contained six putative phosphorylation sites, suggesting that ferritin may be regulated by phosphorylation. These results revealed that phosphorylation could regulate the ferritoid–ferritin interaction and nuclear transport [54]. In the hemolymph, *ScFerHCH* was highly expressed at 6 h, and then decreased at 12 h. However, it could be induced to high expression levels by *S. aureus* at 24 h after injection. Combined with the above bioinformatics and tissue analysis, we speculated that ScFerHCH as a secreted protein might be synthesized in the fat body and transported from the fat body to the midgut or hemolymph. In insects, many proteins show a similar regulatory mechanism. For example, in Drosophila, collagen IV was also synthesized in the fat body and secreted to the hemolymph, and was continuously incorporated into the basement membranes (BMs) of larvae [55].

**Figure 9:**
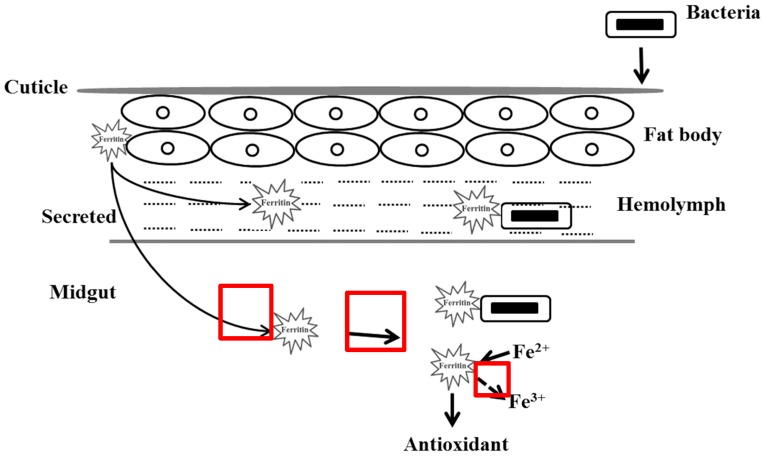
Hypothesized schematic diagram of the H-type ferritin of *S. c. ricini* in the bacterial invasion process. The black arrow indicated direct correlation. The bold black arrow represented clear correlation. The black arrow with dotted line indicated unclear correlation.

Based on the above results, we hypothesized that the roles of ScFerHCH were related to the innate immune response, iron storage, and anti-oxidation in eri-silkworms (Figure 9). Once the bacteria penetrate the cuticle of *S. c. ricini* and enter into the body, ScFerHCH will be synthesized in the fat body and secreted into the midgut and hemolymph. On the one hand, bacteria try to produce the best iron environment for self-growth. However, the ScFerHCH will bind to ferrous ion to reduce the concentration of iron in the hemolymph. On the other hand, excess ferrous ions will produce reactive oxygen according to Fenton’s reaction between ferrous iron and H_2_O_2_, which will activate the anti-oxidation process in eri-silkworms. Taken together, our findings will be useful for understanding the functions of the *S. c. ricini* ferritin heavy chain subunit.

## 4. Materials and Methods

### 4.1. Eri-Silkworm Collection, Bacteria Challenge and Tissue Collection

The eri-silkworm (*S. c. ricini*) B7 strain was obtained from the Sericultural Research Institute of the Chinese Academy of Agricultural Sciences (Zhenjiang, China). One hundred and twenty larvae (fourth-instar molts) were divided into three groups. In each group, the larvae were reared with fresh caster leaves at 27 °C under a relative humidity of 75% and a photoperiod of a 12:12 (Light:Dark).

The overnight cultured *S. aureus* (Gram-positive bacteria) and *P. aeruginosa* (Gram-negative bacteria) cells were harvested and centrifuged at 7500× *g* for 15 min. The collected bacteria were washed three times, and then suspended in sterilized 0.85% NaCl (prepared with sterile water). The third day of fifth-instar larvae were used for bacterial injections. The injections were performed by using microliter syringes (Sangon Biotech, Inc., Shanghai, China) and the wounds were sealed with Vaseline immediately after the injections. The infection groups were injected with 1 × 10^7^ cells of *S. aureus* or *P. aeruginosa* in 10 μL of sterilized 0.85% NaCl, and control groups were injected with 10 μL of sterilized 0.85% NaCl. After the larval infections, eight larvae in each group were dissected to collect the hemolymph, midgut and fat body at 3, 6, 12, 24 and 48 h post-injection. The collected tissues were ground by using liquid nitrogen and stored in TRIzol reagent (Invitrogen, Grand Island, NY, USA) at −80 °C. Each treatment was performed in three biological replicates.

### 4.2. RNA Extraction and cDNA Synthesis

Total RNA was extracted from the hemolymph, midgut, fat body, silk gland, head and integument (different tissues), and hemolymph, midgut and fat body (pathogen challenge) using TRIzol reagent (Invitrogen) according to a previous protocol. The A_260/280_ ratio and RNA concentration of all samples were detected using the NanoDrop 2000 spectrophotometer (Thermo Fisher Scientific, New York, NY, USA). The total RNA samples were treated with the PrimeScript^TM^ RT kit with gDNA Eraser (TaKaRa, Dalian, China) to remove genomic DNA, and then the first strand cDNA was synthesized according to the manufacturer’s instructions. Briefly, 2.0 μL of 5× gDNA Eraser buffer, 1.0 μL of gDNA Eraser and 1.0 μg of total RNA were mixed in a 100 μL PCR tube, then RNase-free H_2_O was added to reach 10 μL, which was then incubated at room temperature for 5 min. Afterwards, 5× PrimeScript buffer, 1.0 μL of PrimeScript RT enzyme mix I and 1.0 μL of RT primer mix were added to the tube, which has made up to 20 μL with RNase-free H_2_O. The mixture was incubated at 37 °C for 15 min, and then incubated at 85 °C for 5 s. The cDNA was stored at −20 °C for later use.

### 4.3. Identification of ScFerHCH from the Transcriptome and Bioformatics Analysis

Transcriptome sequencing was performed on *S. c. ricini* fifth-instar larval hemolymph using the Illumina sequencing method (Beijing Novogene Bioinformatics Technology, Beijing, China) to obtain the *S. c. ricini* transcriptome database [56]. The ferritin heavy chain subunit was identified from the dataset using the TBLASTN algorithm in basic local alignment search tool (BLAST). The candidate ferritin heavy chain subunit was confirmed by searching the NCBI non-redundant protein database using BLASTX (cut-off 1 × 10^−5^). The cDNA and deduced amino acid sequence of ScFerHCH were analyzed using DNAstar and BLAST (http://www.ncbi.nlm.nih.gov/blast). The SignalP4.1 Server (http://www.cbs.dtu.dk/services/SignalP/) was used to predict the presence and location of the signal peptide. The molecular weight (MW) and isoelectric point (pI) of the ScFerHCH protein was calculated by ExPASy (http://web.expasy.org/compute_pi/). Multiple sequence alignments were carried out with DNAMAN 7.0 software (Lynnon Biosoft, Vandreuil, QC, Canada). The phylogenetic tree was constructed with MEGA 5.0 software using the neighbor-joining method with 1000-fold bootstrap resampling [57], and the tertiary structure of the ScFerHCH protein was predicted by phyre online software (http://www.sbg.bio.ic.ac.uk/phyre2/html/page.cgi?id=index). The functional domains were predicted by using SMART software (Available online: http://smart.embl-heidelberg.de/). The iron response element (IRE) in the 5′-untranslated region (UTR) of the ScFerHCH cDNA sequence was predicted by using the SIRES server v2.0 (Available online: http://ccbg.imppc.org/sires/).

### 4.4. Reverse Transcription Quantitative PCR (RT-qPCR) Analysis of ScFerHCH Expression Levels

RT-qPCR was performed to examine the expression levels of *ScFerHCH* in various tissues and under different post-treatments. The primers used in this study were designed by Primer Premier 5.0 (Premier Biosoft, www.premierbiosoft.com) (Table 1). The 25 μL reaction mixture for the RT-qPCR contained 12.5 μL SYBR II, 9.5 μL ddH_2_O, 1.0 μL forward primer, 1.0 μL reverse primer and 1.0 μL cDNA template. The thermal cycling profile consisted of an initial denaturation at 95 °C for 30 s and 40 cycles of 95 °C for 5 s, 60 °C for 30 s, and 72 °C for 20 s. The reactions were conducted in 96-well plates with a Multicolour Real-time PCR Detection System (Bio-Rad, Hercules, CA, USA). Relative expression levels were calculated using the 2^−^^∆∆*C*t^ method [58]. There were three biological sample replicates, and each biological sample replicate included three technique replicates. The reference gene was *S. c. ricini β-actin*. The statistical analysis was conducted using ANOVA and an LSD *a posteriori* test using SPSS (*p* < 0.05).

### 4.5. Prokaryotic Expression and Protein Purification

According to SMART software analysis, we designed primers with restriction enzyme sites of *EcoR I* and *Hind III* at their 5′ end, respectively, to amplify the ScFerHCH ferritin domain (Table 1). The purified PCR product was cloned into pMDTM 19-T Vector (Novagen, Wisconsin, WI, USA) following previous protocols. Positive colonies were selected randomly for DNA sequencing to confirm the correctness of the amplified sequence. Next, the plasmid was extracted, digested, purified and ligated into the pET-28a vector (Novagen). The resulting recombinant plasmid pET-28a-ScFerHCH was confirmed by DNA sequencing, and it was then transformed into *Escherichia coli* BL21 (DE3) (Novagen) competent cells. After being induced by isopropyl β-d-thiogalactoside (IPTG) at 37 °C for 4 h, the cells were harvested by centrifugation at 7500× *g* for 5 min. The cell pellets were suspended in binding buffer (20 mM Tris-HCl, 500 mM NaCl, 5 mM imidazole, pH 7.9) and disrupted by sonication on ice. After centrifugation at 12,000× *g* for 20 min at 4 °C, the recombinant proteins were purified using QIAexpress Ni-NTA Fast Start Kit (Qiagen, Inc, Valencia, CA, USA) according to the manufacturer’s protocol. The quality of purified protein was analyzed by 12% sodium dodecyl sulphate-polyacrylamide gel electrophoresis (SDS-PAGE) and Western blotting.

### 4.6. Antibody Prepartion and Western Blott Analysis

The purified recombinant ScFerHCH protein was submitted to HuaAn Biotechnology Ltd. (HUABIO, Hangzhou, China) for raising rabbit antibody. Briefly, New Zealand White rabbits were immunized with 500 μg of purified proteins homogenized in complete Freund’s adjuvant for three-week intervals. A boost injection of incomplete Freund’s adjuvant was given for another week. Rabbit serum was collected seven days after the last immunization. Monoclonal anti-His antibody (Qiagen, Hilden, Germany) was used to confirm protein expression.

Proteins were extracted from the different tissues of eri-silkworm as previously described [59]. In brief, 100 mg abrasive samples were added to 2 mL centrifuge tubes containing 1 mL of protein lysis buffer (7 M urea, 2 M thiourea, 4% CHAPS). Then, 10 mg of dithiothreitol (DTT) and 1 mM of phenylmethanesulfonyl fluoride (PMSF) were added to 1 mL of lysis buffer before use. The protein extracts (60 μg) were separated on 12% SDS-PAGE gels and transferred onto polyvinylidenedifluoride (PVDF) membranes. The membranes were blocked with 5% non-fat milk in PBST (137 mM NaCl, 2.7 mM KCl, 10 mM Na_2_HPO_4_, 2 mM K_2_HPO_4_, pH 7.5, 0.1% (*v*/*v* Tween-20) for 1 h at room temperature, washed with PBST three times, then incubated with primary antibody (rabbit anti-ScFerHCH (diluted 1:500)) for 2 h at room temperature. After washing, antigen–antibody complexes were detected with a horseradish peroxidase (HRP)-conjugated goat anti-rabbit secondary antibody (1:5000 dilution) (HUABIO) in blocking buffer for 1 h. After another series of washes, immobilized conjugates on the membrane were visualized in HRP substrate solution (Tiangen, Beijing, China). Three biologically independent individuals were performed.

### 4.7. Iron Chelating Assay

The iron chelating capacity of purified recombinant-ScFerHCH was determined based on the method described by Zoysa [60]. Briefly, purified recombinant-ScFerHCH was serially diluted two times to obtain the concentration of 10, 5, 2.5 and 0 mg/mL. Bovine serum albumin (BSA) was diluted and used as a control. Ten microliters of 2 mM FeCl_2_ was added separately into 500 μL of serially diluted protein suspension. After incubation at 22 °C for 10 min, 20 μL of Ferrozine (Sigma, St. Louis, MO, USA) at a concentration of 5 mM was added to the solution and the absorbance at OD_562_ was measured following incubation at 22 °C for 15 min. Three replicates were performed for each assay in this study.

### 4.8. H_2_O_2_ Tolerance Bioassay

The tolerance bioassay of recombinant ScFerHCH to hydrogen peroxide (H_2_O_2_) was conducted according to a previous protocol [61]. In brief, pET28a-ScFerHCH recombinant plasmid was transformed into BL21 (DE3) competent cell, and cultured in LB medium with kanamycin overnight at 37 °C. A single bacterial colony was selected and induced by IPTG as described above. In this study, *E. coli* cells with empty pET28a plasmid were used as a control. The induced cells were then diluted to 0.2 and 0.1 of optical densities (OD) at 600 nm, respectively. Subsequently, a 10 μL droplet of each dilution was plated on LB medium with H_2_O_2_ at a concentration of 0, 2.5 and 5 mM, respectively, and then incubated overnight at 37 °C for. The graph was scanned with a Nikon 7100 camera (Nikon, Surrey, UK).

## 5. Conclusions

In this study, the ferritin heavy chain subunit was identified in the *S. c. ricini* transcriptome database. The cDNA sequence contained a putative signal peptide and a ferrihydrite nucleation center. Gene expression analysis in different tissues revealed that ScFerHCH was highly expressed in the silk gland, fat body and midgut. In response to *P. aeruginosa* and *S. aureus* injection, ScFerHCH was up-regulated at 6 h post-injection. In addition, the recombinant ScFerHCH protein possessed an iron-binding ability and anti-oxidation properties. Together, these results lay the foundation for further research to determine the function of ScFerHCH.

## Figures and Tables

**Table 1:** Primers used in experiments.

Primers	Sequences of Primers (5′ to 3′)	Purpose
F1	GTCTCTACGCCTGCTATCGC	RT-qPCR
R1	GACGCATCCACCTCAGTTTGT	RT-qPCR
β-actin-F2	GGGCCGGACTCGTCATATT	RT-qPCR
β-actin-R2	ATCACAGCCCTCGCTCGCTCCAT	RT-qPCR
F3	CGGAATTC*AAAGACAGCATGCACGC (EcoR I)	Protein expression
R3	CCCAAGCTTTTCCTTGGGTTTGGGAT (Hind III)	Protein expression

* The underline represented restriction enzyme cutting site.

## Abbreviations

RT-qPCR	Reverse transcription quantitative PCR
IBP	Iron-binding protein
HCH	Heavy chain homolog
LCH	Light cgain homolog
IRE	Iron responsive element
BLAST	Basic local alignment search tool
UTR	Untranslated region
IPTG	Isopropyl β-d-thiogalactoside
SDS-PAGE	Sodium dodecyl sulphate-polyacrylamide gel electrophoresis
MSF	Phenylmethanesulfonyl fluoride
PVDF	Polyvinylidenedifluoride
HRP	Horseradish peroxidase
BSA	Bovine serum albumin
ROS	Reactive oxygen species
BM	Basement membrane
